# Rethinking the patient: using Burden of Treatment Theory to understand the changing dynamics of illness

**DOI:** 10.1186/1472-6963-14-281

**Published:** 2014-06-26

**Authors:** Carl R May, David T Eton, Kasey Boehmer, Katie Gallacher, Katherine Hunt, Sara MacDonald, Frances S Mair, Christine M May, Victor M Montori, Alison Richardson, Anne E Rogers, Nathan Shippee

**Affiliations:** 1 Faculty of Health Sciences, University of Southampton, Building 67 (Nightingale), University Road, Highfield, Southampton SO17 1BJ, UK; 2 NIHR Wessex Collaboration for Leadership and Research in Health Care, Southampton, UK; 3 Division of Health Policy and Research, Department of Health Sciences, and the Robert D. and Patricia E, Kern Center for the Science of Healthcare Delivery, Mayo Clinic, Rochester, MN, USA; 4 Knowledge and Evaluation Research Unit, Department of Health Sciences, Mayo Clinic, Rochester, MN, USA; 5 Institute for Health and Wellbeing, College of Medical, Veterinary and Life Sciences, University of Glasgow, Glasgow, UK; 6 University Hospital Southampton NHS Foundation Trust, Southampton, UK; 7 Division of Health Policy and Management, University of Minnesota, Minneapolis, MN, USA

## Abstract

**Background:**

In this article we outline Burden of Treatment Theory, a new model of the relationship between sick people, their social networks, and healthcare services. Health services face the challenge of growing populations with long-term and life-limiting conditions, they have responded to this by delegating to sick people and their networks routine work aimed at managing symptoms, and at retarding – and sometimes preventing – disease progression. This is the new proactive work of patient-hood for which patients are increasingly accountable: founded on ideas about self-care, self-empowerment, and self-actualization, and on new technologies and treatment modalities which can be shifted from the clinic into the community. These place new demands on sick people, which they may experience as burdens of treatment.

**Discussion:**

As the burdens accumulate some patients are overwhelmed, and the consequences are likely to be poor healthcare outcomes for individual patients, increasing strain on caregivers, and rising demand and costs of healthcare services. In the face of these challenges we need to better understand the resources that patients draw upon as they respond to the demands of both burdens of illness and burdens of treatment, and the ways that resources interact with healthcare utilization.

**Summary:**

Burden of Treatment Theory is oriented to understanding how capacity for action interacts with the work that stems from healthcare. Burden of Treatment Theory is a structural model that focuses on the *work* that patients and their networks do. It thus helps us understand variations in healthcare utilization and adherence in different healthcare settings and clinical contexts.

## Background

The idea that illness sometimes involves hard and heavy work is not a new one. The literature on experiences of illness is replete with accounts of people’s struggles to endure the symptoms of illness and to look after themselves and others. The burden of illness and symptoms has been an important focus of this literature [1-3]. Over the past six decades the nature of these burdens has changed, reflecting a new epidemiological and demographic landscape. Where previous generations experienced episodes of infectious and acute disease that were often rapidly lethal because there were few effective treatments, contemporary populations are typically characterized by non-communicable conditions – and thus relationships with health services and treatment modalities – that extend the end-of-life horizon for many years. Importantly, they seem to challenge the solutions currently provided by healthcare systems and policy-makers. Here, major changes in the epidemiological and demographic landscape have led to increasing numbers of people with chronic or long term conditions such as diabetes or asthma; living with and surviving potentially life-limiting conditions, for example, breast cancer, myocardial infarction, stroke; and experiencing degenerative and neuro-degenerative conditions often associated with ageing. These patients exhibit illness trajectories and help-seeking behaviors that healthcare providers and policy-makers perceive as complex and costly, and that seem to represent seemingly uncontrollable demand [4].

The emergence of the ‘chronic’ patient, has been seen in terms of symptom burdens, first in single conditions, and then in the contexts of multiple multi-morbid conditions [5]. But they also experience another kind of burden. This is the burden of treatment itself, as they engage with services and therapeutic modalities aimed at conditions that cannot be cured but must instead be *managed*[6]. This division, over time, between curative effort applied to episodes of acute illness and injury (mainly in hospital), and effort devoted to the management of life-time illness trajectories (mainly in the community) has profoundly changed the nature of both patient-hood and healthcare provision [7].

Management, rather than cure, involves routine work to avoid exacerbation events, detect and avoid recurrence, and to mitigate – and sometimes prevent – disease progression. This is the new *proactive* work of re-engineered patient-hood [8]. Healthcare services increasingly seek to position patients and their supporters as accountable for this work. In turn, this shift in accountability involves adding the *burden of treatment* to the burden of symptoms, as patients experience new and growing demands to organize and co-ordinate their own care, to comply with complex treatment and self-monitoring regimens, and to meet a whole range of expectations of personal motivation, expertise and self-care [7,9-11]. Patients may struggle with the expanding array of tasks expected of them and the resulting burdens, which of course occur alongside the demands of everyday life [12,13]. In turn, this may lead to structurally induced non-compliance and over- or under-utilization of healthcare services as the complexity and weight of these burdens grows over time, as comorbid conditions appear, and as patients’ capacity to meet their demands is overwhelmed [14-20]. As burdens accumulate, and some groups of patients are overwhelmed, the consequences are likely to be poor healthcare outcomes for individual patients, increasing strain on caregivers, and rising demand and costs of healthcare services [9].

The aim of this paper is to rethink what it means to be a patient in the age of chronic multi-morbidity. We need to better understand the resources that patients draw upon as they respond to the demands of both burdens of illness and burdens of treatment, and the ways these resources interact with healthcare utilization. To do this we draw on and integrate outcomes of our previous work. First, Normalization Process Theory [21,22] characterized the processes by which elements of work become embedded in everyday practice, linked this to the problem of patient contributions to the distribution of illness related work [23-25], and informed the development of the concept of *Minimally Disruptive Medicine*[9]. Second, Shippee et al’s., Cumulative Complexity Model [15] outlined relations between the work delegated by healthcare systems to patients (their burden of treatment), and the ways in which they can balance these burdens with capacity to meet the demands of delegated work. The notion of burden of treatment [9,12,14,26,27], has here been useful in conceptualizing the implications of this work. Finally, Rogers et al’s., work on demand, self-care, and social networks [28-31], has emphasized the importance of networks, not just in providing social support, but in distributing and doing important practical work around care.

Our previous empirical and theory-building studies have led us to develop a new model of the relationship between sick people (and members of their social networks) and healthcare services (and their constituent clinicians, administrators, managers, and policy-makers). This model – *Burden of Treatment Theory* – aims to facilitate a new understanding of the interaction between capacity for action and the work that healthcare systems pass on to patients and their relational networks. Importantly, this is a structural model: it helps us understand variations in healthcare utilization and adherence in different healthcare settings and clinical contexts.

## Discussion

In the late 1940s, the American sociologist Talcott Parsons developed a model of the ‘sick role’ that has proven remarkably persistent in medical education and practice. Crucial to Parsons’ model was an individual and private relationship between the patient and a doctor [32,33], that was beginning to crumble even as he set it out. In the intervening period this relationship has been overtaken by a complex network of relationships between patients and providers that are governed by the policies of corporations and governments [34], and in which the supposed inability of many healthcare systems to meet demand has as its corollary in the real inability of many patients to pay for the services that they need. These resulted in an experience of patienthood that is profoundly different to that of fifty, or even twenty, years ago: rationalizing impulses and technological advances in healthcare mean that the nature of patient *and* professional work is changing [35].

Being a patient has come to involve *managed* engagement with multiple healthcare practices that are consequences of the therapeutic revolution of the 1950s and 1960s [36], the emergence of a massive and global biomedical-industrial complex from the 1970s [37], and important developments in the life sciences and technological innovations in measurement and monitoring during the same period [38,39]. These may include complex self-monitoring and treatment regimens, (including widespread polypharmacy), and remote monitoring through telecare and other patient-managed devices [40]. It is underpinned by managerial and behavioral expectations of health behaviors. These emphasize self-care and expert patient regimes, and are founded on ideas about structured self-care, self-efficacy, motivation and engagement [41]. These follow from important political shifts, that have been focused through major debates about the division of responsibility for individual health between government and citizen (in taxpayer funder healthcare systems), and between purchaser and provider (in insurance based healthcare systems).

The degree of accountability that is extended to patients and members of their social networks seems to be a new phenomenon. They are now expected to perform within a set of externally defined parameters: not just in terms of what they do for themselves, but also in terms of the ways that they make demands on services. Indeed, patients are increasingly expected to be more than motivated, but technologically savvy too [42]. Transfers from the clinic to the home have other important consequences. The complexity of some therapeutic regimens means that healthcare becomes the business of whole families and their social networks, but at the same time important professional functions are reshaped. For example, a common strategy is to create a cadre of community nurses whose work focuses on patient surveillance and assessment, and another is to employ non-clinicians who work towards remote management through telecare systems and virtual patient management portals [43]. Service provision is characterized by the intensification of activity for both patients and professionals, as healthcare services seek to do more work, with fewer people, in less time, at lower costs. In turn, this leads to stricter patterns of corporate controls on practice for professionals and patients and thus reshapes the opportunity afforded to patients to engage with health services. The shift to accountability means that the business of being sick involves the patient (and relational network) in a range of tasks that are delegated to them by healthcare systems. With delegation comes a tendency towards defining patients and their relational networks as active ‘partners’, ‘co-producers’, or even ‘co-workers’ in the organization, delivery, and conduct of healthcare work.

### Capacity is a resource to be mobilized

The point of departure for our model is the capacity of individuals and their relational networks to interact with and utilize healthcare services. Here, we focus on patterns of organized and dynamic relations between *agents* (the individuals or groups that interact with each other in relation to healthcare systems), in *contexts* (the diverse technical, professional, and organizational structures that make up healthcare systems and shape opportunities to utilize them).

Here, *agency* refers to the things that people *do* to engage with health problems and with others. The physical, psychological, and sensory dimensions of an injury, disease or disability, or co-morbid combinations of these, have effects on the extent to which a sick person can participate in activities of daily living and the interactions and relationships that sustain them. So do the material and cognitive resources at their disposal. In combination, these have effects on the extent to which people can participate in healthcare services and treatments. The intensity and complexity of these physiological, psychological and social effects may vary over time, limit the extent to which patients can act independently, and may increase their dependence on others [16,25]. Exercising agency may therefore depend on *relationality,* which refers to the social networks through which agency can be expressed and distributed. Unsupported individuals who are isolated from meaningful social networks are not uncommon, especially amongst older people where relational networks are unstable and may diminish towards the end of life [5] but most patients have some kind of mutually supportive social relationships. These may be dyadic (in which one or both persons are sick). They may also take the form of a wider social network (consisting of supportive persons tied together by varying degrees of affective intensity and voluntary or mandatory association and obligation). The intensity, size and complexity of relational networks may vary over time according to the affective and material demands made on members, and the degree of their discretion in meeting these demands [30,44]. Importantly, these relational networks will often include healthcare and other professionals, who may participate and contribute to meeting these affective and material demands. Indeed, their involvement is often mandatory.

Agency and relationality have important implications. First, an individual clinical condition may not be the appropriate unit of analysis. Instead, agency is likely to be inhibited (and dependence promoted) by, for example, the combined effects of multimorbidity and poverty. Second, the appropriate unit of analysis is not necessarily an individual patient, but might be a group of people whose actions compensate (or not) for different kinds of dependence. Of course, these networks do not need to be extensive. However, they may have critical functions in linking to healthcare structures, provider organizations, and professionals. A small number of relational network members may interact intensively over time, building a complex web of interactions across a health economy to secure co-operation and resources from healthcare and social care providers.

Agency and relationality are fundamental, but so too are the properties of the social systems that constrain them. The first of these, *control*, refers to the things that provider organizations do to determine the content of services. Healthcare provision is a corporate activity characterized by attempts to secure the normative standardization of practice (through organizational rules and professional role definitions; clinical guidelines and protocols; technical standards), and the intensification of activity (super-specialization; attempts to improve productivity and cost-effectiveness through changes in organizational structure; new patterns of working; and resource allocation) [35]. Healthcare provision is also characterized by unequal distribution of *opportunities* (defined by the availability of services in different areas and at different times), and by unequal access to services according to clinical status, age, gender, ethnicity and socio-economic status (defined by the structure of the market for healthcare services, and by the explicit and implicit practices of resource allocation within those markets). When healthcare service providers allocate resources and enact policies that determine the distribution of services, they frame opportunities to engage with them.

**Figure 1:**
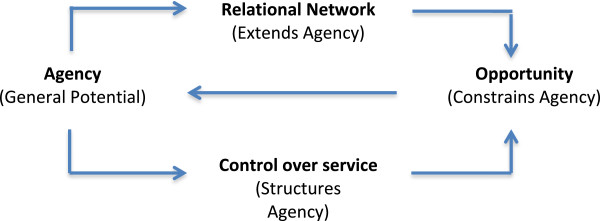
Mobilizing capacity.

The components of capacity that we have so far described (*agency, relationality, control, opportunity*), characterize the relationship between sick people and health services in terms of variations in personal agency and the operation of relational networks; and characterize the relationship between people and health services in terms of variations in opportunity and in the operation of modes of control. At a system level, this can be expressed as a simple diagram, and in Figure 1 we show how agency (the general potential of a patient, or patient group) is mediated simultaneously through their own relational network and through the controls that healthcare providers place on the services that they deliver. These two factors, in turn, shape the opportunities for health care available to the patient, and feed back to structure their potential.

These relationships provide a general structure for healthcare utilization, and for the dynamic interactions between patient capacity and treatment work. Here, the patient’s capacity to engage with treatment work depends on the extent to which they possess agency to participate in this work.

### Capacity and strategic action

Although diagnosing and treating individuals make sense in medical terms – after all, they are the persons who are sick and who must be cared for – the individual patient may not be the appropriate unit of analysis for understanding the dynamics of healthcare utilization. This leads us to a structural model of behavior (individual patient, plus wider social networks including family and other informal support, and networks of specific health and welfare professionals). In this extended unit there may be multiple relationships between network members which offer different degrees of support. Knowledge and beliefs about health and healthcare are often shared, rather than isolated to individuals. Importantly, decisions about what to do, and how to access services, are often distributed amongst multiple participants in a social process [45]. The capacity to accept healthcare work depends on the extent of participants’ abilities to exploit opportunities to utilize healthcare services, and is shaped by the structuring effects of relationality and control.

**Figure 2:**
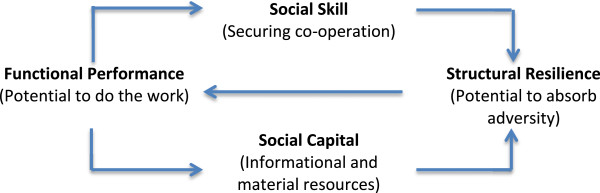
Expressing capacity.

At the granular level of a patient or a group of patients, this model can be expressed as a simple diagram that describes the qualities that patients and their relational networks need to possess if they are to exploit healthcare opportunities. Once again, we express this in a simple diagram (Figure 2). First of all, people who are sick and the people who support them need to perform the material and informational tasks that are asked of them. The *functional performance* of sick people and members of their relational networks refers to the degree to which they possess the cognitive and material capacity to do the things that must be done to meet these demands. The extent to which they possess the necessary *social skill*[46] to engage and mobilize the co-operation of others is central to the construction and maintenance of informal networks. It is also crucial to exploiting opportunities to access healthcare resources and negotiate the controls that are placed on them. It is founded on norms and roles that frame situationally appropriate illness behaviors, interactional strategies, and relationship-building endeavors.

While functional performance and social skill describe the capacity of a patient and relational network to mobilize themselves and others to utilize healthcare services, access to *social capital*[47] is also crucial. This is the extent to which patients and members of their networks can capture, possess, and mobilize membership of the extended social networks through which informational and material resources flow. Following Granovetter [48], this is about the extent to which they can add useful relationships – characterized by weak ties between members – to the core of strong ties through which expectations or obligations of actual material and effective exchange are played out. Finally, we can here consider the question of resilience [49,50]. Typically, resilience is defined in psychological and individual terms, but here, we are also interested in *structural resilience*. By this we mean the extent to which members of the patient’s extended network can capture, possess, and mobilize psychological and social resources to absorb and compensate for – and even thrive – in the face of biographical disruptions [51], adverse path0physiological events and social processes. In Figure 2 we show that the relationship between functional performance and structural resilience is mediated by social skill and social capital, and that resilience feeds back to reinforce functional performance.

The implication of Burden of Treatment Theory is that capacity is not simply a property of individual patients’ functional performance (the limits that the patient’s health and access to socioeconomic resources place on them when they seek to express agency), but it also depends on their – and their relational network’s – social skill (the extent to which they are able to engender co-operation and co-ordination of others) and social capital (the extent to which they are able to access informational and material resources). Thus, improving (or undermining) social skill and social capital affects the extent to which patients and their networks possess structural resilience. The greater the structural resilience of such a network, the more likely it is to be able to compensate for diminishing functional performance over time.

There are limits on capacity in this context, both in terms of the effect of advancing disease on functional performance; and in terms of the extent to which extended relational networks can marshal social skill and social capital to compensate for diminishing functional performance. But it is not just diminishing functional performance that matters here. Limits are also placed on capacity by the uneven distribution of opportunities to engage with healthcare services, and by the controls placed by healthcare providers on the content of those services. The implication of this is that capacity is likely to be highly sensitive to already existing health inequalities. The impacts of socio-economic status, ethnicity, age, and gender on both gradients of health status and access to services are well established and incontrovertible [52-54]. We have previously argued that the illness careers of people with long-term conditions are characterized by cumulative complexities that arise from interactions between patients and healthcare providers [15] as they experience the changing relationship between capacity and work. Against the background of a structural model of capacity, we might expect that over time interactions between patients (whose capacity may be diminishing, and whose relational networks may be less able to compensate for this), and their opportunities to utilize healthcare services (which are reduced as unmet dependencies increase) are characterized by relative degrees of cumulative disadvantage.

### The structure and performance of patient work

Having characterized some of the key aspects of capacity, we can now turn to the question of work itself. Normalization Process Theory can help us to identify the domains of work that make possible the routine incorporation of patient work into everyday life. In this context, we can see the work of the patient, or indeed the doctor and nurse, in terms of four generative mechanisms and their necessary investments. These mechanisms are expressed through four kinds of patient work [24,25,55].

At a system level [56], these categories of work include individual and collective *sense-making* in which sick people and members of their social networks are expected to identify, understand and explain the diverse tasks that make up their work, and to internalize and plan for their requirements. The more complex and demanding work is, the more likely it is that sick people and members of their relational networks will need to invest in enrolling others into it, and initiating and sustaining work that focuses on network formation and co-ordination of *cognitive participation*. Because relational networks are placed under strain as obligations are distributed within them, it involves members continuously investing in network maintenance. Sense-making and participation are fundamental requirements for *collective action.* Sick people and members of their relational networks are allocated and execute specific tasks, negotiate accountability for their outcomes, and organize and realize the mobilization of resources that make them possible. This requires them to invest in *doing* symptom management and service coordination. But they must also be engaged in *reflexive monitoring.* Sick people and members of their social networks engage in the systematic collection of information about signs and symptoms and about the views of significant others, undertake its individual and collective appraisal, and apply it to the reconfiguration of their work.

**Figure 3:**
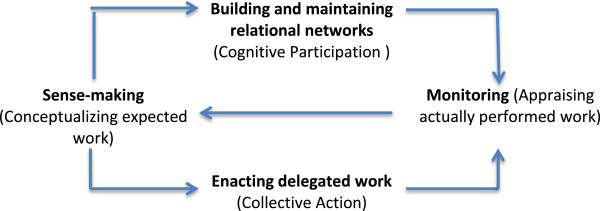
Mobilizing for delegated tasks.

**Figure 4:**
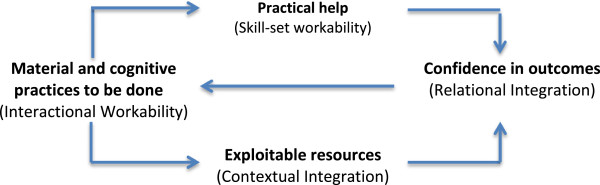
Enacting delegated tasks.

The relationship between these four constructs: *sense-making*, *cognitive participation*, *collective action, reflexive monitoring* is described in Figure 3. They refer to important elements of work as we can conceptualize it at a systemic level, but at the level of individual patients or patient groups and their relational networks we might expect to find a more granular set of practices that structure collective action [57]. These are set out in Figure 4, where the *interactional workability* of delegated practices matters. Where patients and their relational networks cannot do the work, because it has material or cognitive requirements that are beyond them, because devices cannot be made to work, or because the work itself has adverse consequences for the patient (or for members of the relational network), then the whole enterprise is under threat. But of course, if delegated work is interactionally workable – and if patients and their relational networks possess both the practical skills (*skill set workability*) and local exploitable resources (*contextual integration*) to make it work – then there is a high probability that delegated work will become routinely embedded in everyday practice. One further factor may promote or inhibit this, and this is *relational integration*: the extent to which patients and members of their relational networks have trust in delegated tasks and confidence in their outcome.

The burden of work, here, refers not just to the weight of specific tasks, but also to the weight of implementing and maintaining them alongside the demands of other aspects of everyday life. This is done in the context of holding together something much larger and more complex than merely complying with treatment instructions, maintaining a set of self-care activities, and holding on to a set of organizational expectations. For many people, these are large scale social accomplishments that involve no less complicated business relationships and intensification of activity than those experienced by health professionals in practice. It can be hard and heavy work.

### Instability is normal

Our model characterizes a set of mechanisms through which agency and work are expressed and enacted. These form fundamental conditions within which illness careers and disease trajectories are experienced. We have already observed that symptoms (and treatments) of many diseases affect functional performance. Such symptoms often include fatigue or other impairments that mean that patients rely on their relational networks as sources of *prosthetic* agency. In advanced disease (for example, primary brain cancers, Alzheimer’s disease, and end-stage COPD) there may be a complete transfer of agency and accountability from the patient to members of the relational network.

Relational networks are inherently unstable, because their achievements are accomplished day by day in competition with other commitments, and because of variations in social and material resources [58]. Their memberships change. They may expand or contract. Relations within them may become more or less complex, more or less supportive, and exchanges of information and services within them more or less efficient [30,59]. Networks may degrade over time because of the strain of work that is distributed to them, or because members are demoralized by the course and effects of disease itself. They may collapse because of successive exogenous shocks, including the sickness, departure, or death of members. Degradation and collapse may occur rapidly (for example, amongst people with lung cancer, where support groups may be characterized by high mortality), or over extended periods of time (for example, because of the compounding effects of age, associated with health problems that affect both physical and cognitive integrity, and also impact on the size and capability of social networks).

Patient and relational networks are fragile. The closure of a bus route, the loss of a job or car, or the relocation of a clinical facility to another suburb or city, may destabilize a relational network and undermine its structural resilience. The production of capacity, and the implementation and embedding of delegated tasks, must then begin again. Disease trajectories and relational instabilities mean that the burden of treatment must be continually reproduced.

### Burden of Treatment Theory: how do capacity and work interact?

So far, we have treated capacity and work as conceptually distinct properties of a social system. Both extend far beyond the transaction spaces of the clinic. They represent highly complex, variable, and emergent behaviors of both patients and members of their relational networks, and the healthcare systems and professionals that that they engage with. They vary, too, according to aspects of the condition or conditions that they respond to: collective agency, healthcare systems, patients’ behaviors, and investments in work are very different when the patient is depressed, or when the patient has end-stage astrocytoma. The social and economic resources available to sick people matter very much – and this includes the numerical strength and resource richness of their relational network.

Against this background, a useful lesson of research on complex systems is that complexity arises out of what often seem to be simple interactions and rules [60,61]. Research on the structures of theoretical explanation [62], suggests that the most robust and efficient conceptual models tend to focus on a relatively small set of strong primary assumptions about the dynamics of behavior within systems. In this paper we have aimed to present a minimum set of strong primary assumptions that draw on robust empirical and theory-building research. We have chosen not to discuss the multiple contingent factors that are known to affect the ways that sick people interact with healthcare services. Focusing on generative principles [63] means that we can put aside these factors – which constitute the contingent periphery of explanations – in favor of a set of general and generalizable assumptions about the dynamics of behavior within healthcare systems and of the relationships between capacity, work and healthcare utilization. These are that:

∎ At a societal level, illness and healthcare utilization are social experiences characterized by social networks that are meaningful and significant to participants. They are governed by expectations of accountability and norms of membership and behavior. These give structure to social relationships and interactions that constitute healthcare utilization as a social system, and define the necessary degree of competence of participants.

∎ At a system level, patients and their relational networks can act as collective agents to negotiate and navigate healthcare services. Their exercise of agency is constrained by controls on service content and the distribution of opportunities for care, and by the social and economic resources available to participants. Experiences of these constraints reinforce or change behaviors. *Interventions that interventions that build and strengthen relational networks around sick people, and that equip them to more effectively navigate system controls and opportunities, are therefore likely to improve effective healthcare utilization.*

∎ At a system level, patients and their relational networks can act as collective agents to conceptualize expectations about behaviors and tasks, to build and reinforce social networks, enact delegated tasks, and appraise the effects of these processes. Experiences of these effects reinforce or change behaviors. *Interventions that facilitate work to secure co-operation and social capital and so compensate for deficiencies in functional performance and improve structural resilience are therefore likely to increase capacity to take on delegated healthcare tasks.*

∎ At a granular level, patients and their relational networks can act as collective agents to possess the ability to perform the multiple tasks that are transferred to them by healthcare systems, to secure the co-operation of others, and to add to their social capital. Experiences of these reinforce or change structural resilience. *Interventions that facilitate controls on the load of cognitive and practical tasks delegated to patients and their relational networks, and that monitor their effects, are therefore likely to improve capability to perform delegated healthcare tasks.*

∎ At a granular level, patients and their relational networks can act as collective agents to invest in work to perform material and cognitive tasks, invest in the skills that must be distributed amongst them, identify and exploit local resources, and consider the outcomes of this work. Experiences of these reinforce or change confidence in the tasks that they have been delegated. *Interventions that maximize collective competence in enacting practical tasks, distributing help and exploiting local resources, and effect increased confidence in healthcare processes and outcomes, are therefore likely to reduce inappropriate demands on healthcare services.*

**Figure 5:**
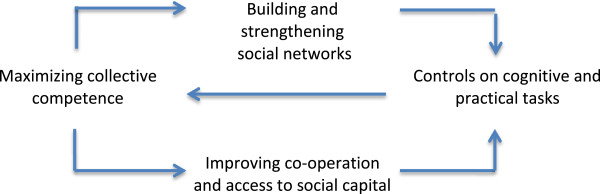
Interventions that link capacity and work.

∎ Agency and work are unstable situational accomplishments, and interactions between patients and relational networks are affected by multiple endogenous and exogenous factors. Functional performance and structural resilience are vulnerable to instabilities and responses to the burden of treatment must be continuously reproduced.In Figure 5, we show how these interventions are likely to be arranged in practice. Improving the quality and effectiveness of collective action lifts the burden from individuals. After all, healthcare policy-makers are anxious about demand management, and claims of patient partnership are often linked to policies of ‘self-care’ or ‘supported self-management’ that are intended to reduce engagement with formal health services and hold patients at a distance. These assumptions characterize a set of social processes in which participants need to be highly skilled at assembling and utilizing collective resources. Crucially, they tells us why some people fail to get the best – or sometimes anything at all – from healthcare services, while others are able to garner support from their social networks and healthcare system that successfully sustains them until the end of life. All of this is underpinned by the acknowledgement that almost every aspect of sickness and engagement requires investment in complex relational and practical tasks. The patient, in contemporary healthcare, is an active part of the system – whether they like it or not.

## Summary

Conceptual models and theories abound in health care [64]. We need to move beyond program theories and connect analytic models with practice initiatives. To this end, in an earlier paper, some of us called for *minimally disruptive medicine*[9] as a response to the work that is delegated to patients and their families. We argued that by redesigning healthcare services so that they are better coordinated and more patient-centered in their delivery of services, and so that they acknowledge patient complexity and patients’ preferences, patients could be better equipped to handle their health problems. Minimally disruptive medicine involves major changes in thinking about how ‘whole systems’ function and what they do. Critically, it involves respecting patients for what they do, as well as for who they are.

Practice changes often flounder in the face of the complexities of organizational inertia and professional resistance, and change management is a major problem when large scale institutional and professional interests are at stake [65]. This has been amply revealed by recent policy debates in the United States and United Kingdom about the organization and funding of healthcare provision. Even relatively restricted changes in the organization of clinical practice can lead to a battery of unanticipated consequences and perverse incentives [66]. The key question here is about the strategic direction of healthcare services: what kinds of changes are necessary to improve patient experiences of complex and cumulative burdens? Burden of Treatment Theory suggests that interventions that will improve patient experience are those that acknowledge and attack dysfunctional structural elements of healthcare utilization. Such interventions could make a real difference to the ways that sick people and their relational networks utilize healthcare services.

Across the developed world, policy and practice increasingly focuses on developing the ‘self-actualizing patient’ and stresses self-management and self-care [67]. Such approaches often seek to improve motivation and ensure compliance, when the resources to achieve these ends are often simply not available to individual patients. While further work needs to be done to refine and validate this theoretical model, it is clear that rethinking the patient calls for actively investing in improving capacity and managing workload in order to promote better experiences of illness, more effective healthcare utilization, and better healthcare outcomes.

## Competing interests

The authors declare that they have no competing interests.

## Authors’ contributions

CRM authored and is guarantor of this paper, which is the result of a series of discussions between the authors from 2010 through 2013. CRM, VMM and FSM led the conceptual work leading to the paper. All authors made important contributions the paper, made multiple revisions of the manuscript for important intellectual content, and gave final approval of the version to be published.

## Pre-publication history

The pre-publication history for this paper can be accessed here:

http://www.biomedcentral.com/1472-6963/14/281/prepub
